# Isolation and Genetic Characterization of *Mother-of-Snow-White*, a Maternal Effect Allele Affecting Laterality and Lateralized Behaviors in Zebrafish

**DOI:** 10.1371/journal.pone.0025972

**Published:** 2011-10-13

**Authors:** Alice Domenichini, Marco Dadda, Lucilla Facchin, Angelo Bisazza, Francesco Argenton

**Affiliations:** 1 Dipartimento di Biologia dell'Università degli Studi di Padova, Padova, Italy; 2 Dipartimento di Psicologia Generale dell'Università degli Studi di Padova, Padova, Italy; University of Otago, New Zealand

## Abstract

In the present work we report evidence compatible with a maternal effect allele affecting left-right development and functional lateralization in vertebrates. Our study demonstrates that the increased frequency of reversed brain asymmetries in a zebrafish line isolated through a behavioral assay is due to selection of *mother-of-snow-white* (*msw*), a maternal effect allele involved in early stages of left-right development in zebrafish. *msw* homozygous females could be identified by screening of their progeny for the position of the parapineal organ because in about 50% of their offspring we found an altered, either bilateral or right-sided, expression of *lefty1* and *spaw*. Deeper investigations at earlier stages of development revealed that *msw* is involved in the specification and differentiation of precursors of the Kupffer's vesicle, a structure homologous to the mammalian node. To test the hypothesis that *msw*, by controlling Kupffer's vesicle morphogenesis, controls lateralized behaviors related to diencephalic asymmetries, we analyzed left- and right-parapineal offspring in a “viewing test”. As a result, left- and right-parapineal individuals showed opposite and complementary eye preference when scrutinizing a model predator, and a different degree of lateralization when scrutinizing a virtual companion. As maternal effect genes are expected to evolve more rapidly when compared to zygotic ones, our results highlight the driving force of maternal effect alleles in the evolution of vertebrates behaviors.

## Introduction

During the evolution of animal body plans, symmetry has been broken at least three times, possibly for purposes linked to feeding and escape behaviors. It can be easily recognized that the last broken symmetry is the left-right (also called bilateral) whose level ranges from extreme situations (e.g. *Solea spp*) to the almost perfect external appearance of many animals. From the evolutionary point of view it can be supposed that levels of bilateral symmetries can be limited or promoted by natural selection due to the social and individual trade offs associated with survival and reproduction [1], [2], [3]. In vertebrates, the bilateral symmetry of the external body plan conceals consistent asymmetries in the disposition, morphology and function of internal organs. Genetic and molecular mechanisms that establish LR identities of the two halves of the developing embryo act between late gastrulation and early somitogenesis and are known to be conserved amongst the different classes of vertebrates [4]. In the vertebrate embryo the event responsible for breaking initial symmetry occurs during late gastrulation at the posterior end of the notochord in an evolutionarily conserved transient ciliated structure: the mammalian node, the gastrocoel roof plate in Xenopus; the Kupffer's vesicle in zebrafish [5], [6]. Cilia of this structure displays a rotating beating movement that generate a leftward flow of extracellular fluid which triggers the asymmetrical transcription of Nodal genes on the left lateral plate mesoderm [7], [8], [9]. These mechanisms ultimately result in the expression of genes of the *nodal* and *lefty* families and, subsequently, to that of the transcription factor pitx2 in the left lateral plate mesoderm (LPM) of chick, mice, frog and zebrafish [10], [11]. Disturbance or absence of nodal leftward flow results in laterality defects and randomization of left-right asymmetries in vertebrates and *situs inversus* in humans [12], [13], [14], [15], [16]. Recently it has been reported evidence of *nodal* and *Pitx* orthologues expression in two species of snails with opposite body handedness and direction of shell coiling. Authors found that *nodal* and *Pitx* are both expressed in the embryo on the right side in dextral species and on the left side in sinistral species. These results suggest that the asymmetrical expression of *nodal* and *Pitx* may represent an ancestral feature conserved in the evolution of Bilateria [17]. Furthermore, from previous studies it is known that in snails, body handedness is controlled by a maternal effect trait that determines the direction of shell coiling in the offspring [18], [19], [20].

In a recent work, we have observed that two lines of zebrafish selected for opposite behavioral lateralization, also showed differences in anatomical left-right asymmetries [21]. Using the mirror test (a test in which animals could observe their own reflections recognized as a social reward [22]), we also observed that opposite selection in two fish lines (GTLE, fish selected from the wt strain Giotto Leo, with a bias in left-eye use and TLRE, selected from the wt strain Tupfel Longfin, with a preference for right eye use), could increase the frequency of individuals lateralized in a specific direction, while decreasing the frequency of individuals lateralized with the opposite eye preference. We also showed that selection for right-eye preference in inspecting a social stimulus increased the frequency of individuals with reversed epithalamic asymmetries; in the TLRE strain, after five generation of artificial selection the frequency of embryos with reversed asymmetry in the position of the parapineal organ increased from 12.5% of the wild type stock (TL) to 35.8% [21]. Thus, results of the work suggested that behavioral asymmetries could have a genetic basis in zebrafish and that their selection can lead to a rapid change in neuroanatomical and behavioral phenotypic frequencies.

At least two more studies provided evidences of a correlation between neuroanatomical, visceral and behavioral asymmetries. Barth et al. [23], studying the mutant *frequent situs-inversus* (*fsi*) line, found that adult zebrafish with normal heart position preferred to bite targets on their right, while fish with reversed heart position did the opposite. Moreover, fry with either right or left heart were found to have an opposite pattern of eye use in the mirror test, although they did not differ in two other lateralized measures. Recently, Dadda et al. [24] observed adult zebrafish, sorted for right or left parapineal position, in a series of assays that measure visual and motor laterality and found significant differences between fish with opposite parapineal position in all laterality tests used.

In this study we have continued the genetic analysis of the alleles isolated by Facchin et al. We found that in zebrafish a polymorphic maternal effect variant called *mother-of-snow-whit*e (*msw*), affects morphogenesis of Kupffer's vesicle and controls the proportion of LR asymmetries in the progeny. A second purpose of this work was to evaluate the role of the msw maternal allele in the development of neuroanatomical asymmetries which seem to be associated with behavioral asymmetries. To establish whether a concordance exists between parapineal position and eye use we compared adults with opposite parapineal position in a modification of the “viewing test” used by Facchin et al. [25], to estimate asymmetries in eye use. Our results show that in zebrafish, a maternal effect gene polymorphism controls both laterality and lateralization by means of a conserved genetic expression cascade, highlighting the power of maternal effect genes in the evolution of animal behavior.

## Results

### Genetic analysis of diencephalic asymmetries in GTLE and TLRE strains

Facchin et al. could isolate in five generations two strains of zebrafish: GTLE and TLRE that used preferentially the left or the right eye, respectively, when inspecting a mirror. Histological analysis of larvae of the two strains revealed that TLRE fish had a very high percentage of larvae with parapineal on the right side of the body [21]. In the present work we decided to perform selective crosses in order to assess the genetics underlying the trait selected by Facchin et al. We first analyzed the offspring from reciprocal crosses using fish from the 5th generation of selected lines. In the first cross females of the GTLE line (with a preference for left-eye) were paired to males of the TLRE line with the opposite preference, and in the second cross, females of the TLRE line were paired to males of the GTLE line.

**Figure 1:**
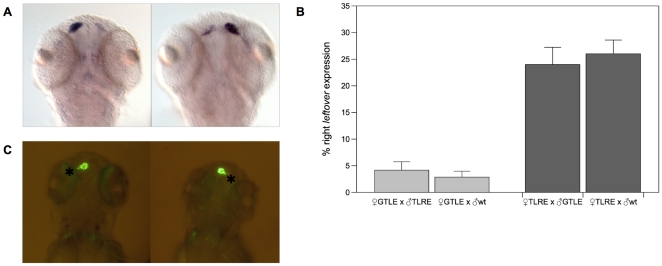
Zebrafish brain asymmetries are reversed in embryos from TLRE line. **A,** *in situ hybridization* on 3 dpf embryos showing the expression of the *leftover* (*lov*) gene, a marker of habenular L-R asymmetries. Normal *lov* expression is stronger in the left habenular nucleus, while in larvae with reversed asymmetries *lov* expression is stronger in the right habenula. **B,** frequencies of embryos with reversed (right) *lov* expression in embryos derived from GTLE females mated TLRE or wt males males and from TLRE females mated to GTLE or wt males. **C,** *in vivo* detection of the position of the parapineal organ (asterisk) in transgenic tg(*foxD3*:GFP)zf15 zebrafish. GFP expressed in the pineal complex allows discriminating between left parapineal (LEFT PPO) and right parapineal (RIGHT PPO) fish.

For each reciprocal cross (Table 1), embryos were collected and at 3 dpf we scored the position of diencephalic asymmetries by detection of *leftover* expression. The expression of this habenular marker has been reported to be stronger in the left dorsal habenula than in the right in about 95–97% of the embryos. This is a consequence of the neural connection of the left-sided parapineal organ with the ipsilateral habenular nucleus [26]. Conversely, when diencephalic asymmetries are reversed and the parapineal organ is on the right side of the epithalamus (about 3–5% of wild type embryos), the expression of *leftover* is stronger in the right habenula [26], [27] (figure 1A). Thus, larvae resulting from reciprocal crosses have been classified for the position of their parapineal organ and scored either as left sided (L-PPO) or right sided (R-PPO). Four different GTLE females were mated to TLRE males and, as a result, embryos with right-sided parapineal organ (R-PPO) were (mean±SD) 4.3%±2.3 of their offspring (n = 460 embryos from 4 females). This frequency is similar to that found for wild type strains reported in literature (Chi-square = 0.256; p = 0.613 [26]). The same result has been observed also when GTLE females were crossed to WT males (two-sample t test, t_(6)_ = 0.391, p = 0.709): embryos with reversed brain asymmetries (R-PPO) were in mean 3.6%±2.6 (n = 441). Conversely, mating pairs between four different females from TLRE line and males from GTLE produced offspring in which a mean of 23.9%±5.6 of embryos showed reversed epithalamic asymmetries (n = 699). The same frequencies of R-PPO offspring were reported when TLRE females were crossed to males of wild type strains (mean 26.1±4.5; n = 706). No significant difference has been found between the crosses of TLRE females either with GTLE or WT males (two-sample t test, t_(6)_ = 0.575, p = 0.586) (figure 1B). Moreover when WT females are mated to either GTLE or WT males the frequencies of reversed asymmetries are respectively 4.0%±3.5 and 4.0%±2.0 (two-sample t test, t_(4)_ = −0.014, p = 0.990) (see Figure S1).

**Table 1:** Reciprocal crosses between males and females of the two selected GTLE and TLRE.

♀GTLE x ♂ TLRE	n	left *lov* 1	right *lov* 1	% right *lov* 1
♀1×♂3	93	89	4	4.3
♀2×♂3	96	94	2	2.1
♀3×♂1	30	29	1	3.3
♀6×♂2	179	162	17	9.5
♀6×♂3	62	61	1	1.6
**♀GTLE×♂wt**				
♀1×♂1	132	124	8	6.1
♀2×♂2	30	30	0	0
♀2×♂1	36	36	0	0
♀3×♂3	69	66	3	4.3
♀3×♂5	15	15	0	0
♀4×♂3	82	78	4	4.9
♀5×♂2	63	60	3	4.8
**♀TLRE×♂GTLE**				
♀1×♂1	115	97	18	15.65
♀2×♂2	288	209	79	27.4
♀3×♂3	164	122	42	5.6
♀4×♂4	132	96	36	27.3
**♀TLRE×♂wt**				
♀2×♂1	36	25	11	30.5
♀1×♂2	201	150	51	25.4
♀5×♂3	189	151	38	20.1
♀4×♂4	332	239	93	28.0

1 *leftover* espression detected by in situ hybridization.

The table shows the results from mating different GTLE females with different TLRE and wt males, and TLRE females with different GTLE and wt males. Only TLRE females could generate a higher frequency of larvae with reversed parapineal organ, independently from the male. n = number of embryos per each cross; left *lov* = number of embryos with stronger expression of *leftover* in the left habenula; right *lov* = number of embryos with stronger expression of *leftover* in the right habenula.

These results led to the most suitable hypothesis that the behavioral selection promoted the isolation of a spontaneous polymorphism in a maternal effect gene that we called *mother of snow-white* (*msw*) after the fact that the maternal allele that controls the offspring phenotype has been selected using the mirror test.

**Figure 2:**
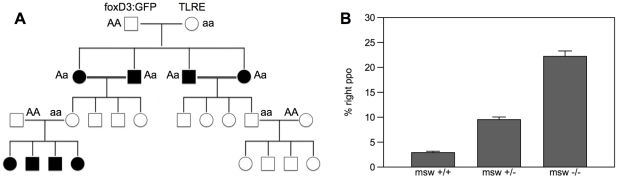
*Msw* allele is a maternal effect gene determining laterality in zebrafish. **A,** pedigree that shows the inheritance predicted for a maternal effect gene. **B,** percentage of reversed parapineal position for the three groups of females classified after the genetic analyses. Mean±SE are expressed.

In order to validate this hypothesis, selected TLRE females were mated to transgenic males of the Tg(foxD3::GFP)zf15 line (F_0_), in which GFP is expressed also in the pineal complex thus allowing the *in vivo* scrutiny of the position of the parapineal organ [28] (figure 1C). Larvae from F_1_ generation were screened for the position of the parapineal organ at 3 dpf and fish with left (72.0%; n = 239/332) and right (28.0%; n = 93/332) parapineal organ were raised separately. Then, F_1_ males and females with right or left parapineal were mated to obtain the F_2_ generation (figure 2A). Once raised to adulthood, F_2_ females were mated to different wild type males and their progeny has been screened for the position of the parapineal organ to identify homozygous recessive *msw* female carriers.

We analyzed 83 females each undergoing at least two distinct mating events with different males. When collecting data from females' progenies, we designate three genotypic classes based on the frequency of R-PPO embryos produced by each female: *msw* ^+/+^ if R-PPO≤5%; *msw* ^+/−^ if 5%<R-PPO<16%; *msw* ^−/−^ if R-PPO≥16%; (figure 2B).

Twenty-four *msw* ^+/+^ females produced a mean of 2.9%±1.3 R-PPO over 16226 embryos; thirty-nine *msw* ^+/−^ females produced a mean of 9.5%±3.3 R-PPO over 27095 embryos and twenty *msw* ^−/−^ females produced a mean of 22.2%±4.8 R-PPO over 12742 embryos. Thus, the three classes significantly differed in the percentage of reversed parapineal offspring (F_(2,83)_ = 184.812, p<0.001). For simplicity embryos derived from *msw* ^?/?^ females will be referred to as M-*msw* ^?/?^ embryos.

### Analysis of asymmetric gene expression in embryos resulting from msw^−/−^ females

**Figure 3:**
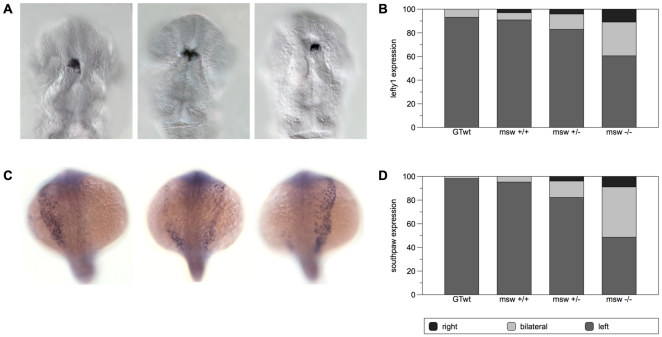
*Msw* allele randomize Nodal pathway. **A,** From left to right, dorsal view of left-sided, bilateral and right-sided expression of *lefty1* in the dorsal diencephalon detected in 22 somite-stage embryos. **B**, Percentages of normal (left-sided), bilateral and reversed *lefty1* in WT control (n = 44), embryos from *msw^+/+^* females (n = 33), embryos from *msw^+/^* ^−^ females (n = 435) and from *msw* ^−*/*−^ females (n = 403). **C,** *spaw* expression in the dorsal diencephalon in 15–18 somite-stage embryos. From left to right, dorsal view showing normal (left-sided), bilateral and right-sided expression. **D,** Percentages of normal (left-sided), bilateral and reversed *spaw* in WT control (n = 169), *msw^+/+^* females (n = 126), embryos from *msw^+/^* ^−^ females (n = 203) and from *msw* ^−*/*−^ females (n = 753).

We next examined the transcription of the asymmetric marker *lefty1*, which is expressed in the dorsal diencephalon before development of epithalamic structures [26], [29] (figure 3A). Results show that a reduced percentage of M-*msw* ^−/−^ embryos had normal left-sided *lefty1* expression (60.5%) compared to M-*msw* ^+/+^ and M-*msw* ^+/−^ (figure 3B). The three genotypic classes were significantly different in their offspring phenotypic frequencies (Chi square 59.801; p<0.001) while M-*msw* ^+/+^embryos do not significantly differed from wild type (Chi-square = 1.361; p = 0.506).

Based on these results, we decided to focus on a previous step in zebrafish bilateral development, the expression of the gene *southpaw* (*spaw*), the earliest nodal-related gene with a LR asymmetrical expression described in zebrafish (figure 3C). *spaw* is transcribed along the left lateral plate mesoderm (LPM) and predicted to promote the expression nodal-related genes, *cyclops* (*cyc/ndr2*) and *lefty1* in the left dorsal diencephalon during later somitogenesis stages [30]. Results for the expression of *southpaw* in the LPM in 15-18-somite stage embryos although similar to those of *lefty1* expression in the left dorsal diencephalon (figure 3D), show a more extreme polarization of frequencies of embryos from females of the three different genotypes, with only 48.6% of M-*msw* ^−/−^ embryos having normal left-sided spaw expression. As for *lefty1* expression, the three classes were significantly different (Chi square 148.659; p<0.001) while M-*msw* ^+/+^embryos do not significantly differed from wild type (Chi-square = 2.139; p = 0.144).

### Genetic analysis of Kupffer's vescicle morphology

The activation of *spaw* expression has been shown to occur after the onset of the cilia-driven leftward fluid flow generated by the zebrafish Kupffer's vesicle (KV) [13]. Evidence provided on *spaw* expression in M-*msw* ^−/−^ embryos suggested that the characterization of this maternal effect allele may extend to earlier developmental stages. Elegant studies demonstrated how disrupted leftward flow affects the normal left sided expression of *Nodal* in the left LPM [13], [31], [32], we therefore decided to analyze this event in *msw* ^−/−^ embryos. Furthermore it has been shown that events disrupting normal Kupffer's vesicle morphogenesis also lead to laterality defects [33]. Considering these evidences, we first analyzed the morphology of KV in embryos derived from *msw* females. In line with a previous study on the morphological characterization of the zebrafish Kupffer's vesicle [34], we focused on the major cross-section to measure the antero-posterior (AP) and left-right (LR) diameters as well as the total area of Kupffer's vesicle.

**Figure 4:**
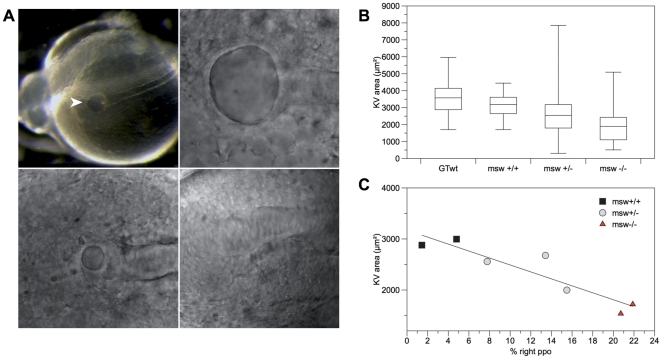
*Msw* allele influence KV morphogenesis. **A,** zebrafish wt embryo at the 10-somite stage. Kupffer's vesicle facing upwards (white arrowhead) is visible at the tail bud at the end of the notochord (n). The other panels show normal, reduced, and no Kupffer's vesicle in embryos at the 10-somite stage. Scale bar = 50 µm. **B,** measures of area of KV in wt control embryos and in embryos derived from females of the three analyzed classes expressed as box plot (whiskers represent smaller and larger values for each group). Mean±SE are expressed. **C,** significative reverse correlation between the size of KV of embryos derived from two *msw^+/+^,* three *msw^+/^* ^−^, and two *msw* ^−*/*−^ females and the frequency of larvae with reversed brain asymmetries generated by the same females.

Results showed a significant reduction in the size of KV of M-*msw* ^−/−^ embryos (in detail, a 25.8% reduction in AP diameter and a reduction of 24.3% in LR diameter), in addition, a percentage of 12.5% of M-*msw* ^−/−^ embryos completely lacked Kupffer's vesicle (figure 4A). We could not detect wild type or M-*msw* ^+^ embryos lacking KV. The AP average diameter is 48.6±14.3 µm in M-*msw* ^−/−^ embryos (n = 39); 57±13.0 µm in M-*msw* ^+/−^embryos (n = 206) and 65.5±7.7 µm in M-*msw* ^+/+^ embryos (n = 25). The difference between the three maternal genotypic classes of embryos is statistically significant (F_(3,376)_ = 34.08, p<0.001). Post hoc analyses (LSD method) revealed no significant difference between WT control (n = 110) and M-*msw* ^+/+^ embryos (p = 0.291).

The LR average diameter is 50.5±13.6 µm in M-*msw* ^−/−^ embryos (n = 39); 58.6±12.4 µm in M-*msw* ^+/−^embryos (n = 206) and 66.7±9.2 µm in M-*msw* ^+/+^ embryos (n = 25). Again, the difference between the three maternal genotypic classes of embryos is statistically significant (F_(3,369)_ = 34.65, p<0.001). Post hoc analyses (LSD method) revealed no significant difference between WT control (n = 104) and M-*msw* ^+/+^ embryos (p = 0.287).

Finally, the calculated area of KV in M-*msw* ^−/−^, M-*msw* ^+/−^ and M-*msw* ^+/+^ embryos is 1903.3±1061.8 µm^2^ (n = 38), 2565.3±1051.5 µm^2^ (n = 205) and 3156.8±695.5 µm^2^ (n = 25). Differences in the size of KV between the three maternal genotypic classes of embryos (figure 4B) is highly significant (F_(3,368)_ = 36.10, p<0.001), but post hoc analyses (LSD method) revealed no significant difference between WT control (n = 104) and M-*msw* ^+/+^ embryos (p = 0.074). A correlation analysis has also been performed to verify if there was a correspondence between the percentage of embryos with reversed parapineal produced by females of the three classified genotypic classes and the size of KV of their offspring. We analyzed KV and brain asymmetries of embryos and larvae respectively derived from two *msw* ^+/+^ females, three *msw* ^+/−^ females and two *msw* ^−/−^ females. As reported in the graph (figure 4C) a significant negative correlation has been found, as when the area of the Kupffer's vesicle increases, the percentage of embryos with reversed parapineal decreases (Pearson correlation r = 0.565).

Considering these results on KV morphology, we aimed at verifying whether *msw* ^−/−^ allele, reducing KV size, should also affect (blocking or at least reducing) the directional flow generated by cilia-beating into the KV in M-*msw* ^−/−^ embryos, thus leading to a randomization in the expression of *nodal-related* genes and *nodal*-dependent downstream genes. Beads microinjection procedure [13], [35] was performed on embryos derived either from wild type or *msw* ^−/−^ females. We successfully injected 15 M-*msw* ^−/−^ embryos, all showing a counterclockwise movement of fluorescent beads inside the lumen of KV. Similarly 25 control embryos have been successfully injected all showing a counterclockwise movement of fluorescent beads inside the lumen of KV (Movies S1 and S2).

**Figure 5:**
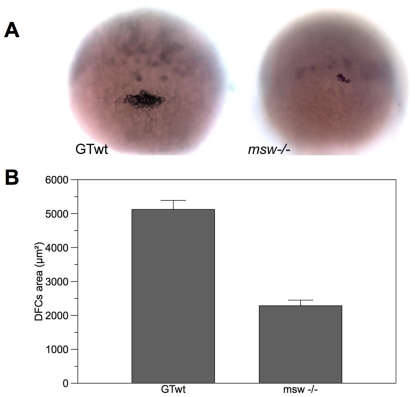
*Msw* allele seems to affect DFCs differentiation. **A,** *sox17* expression at 50% epiboly in wt (left) and *msw* ^−*/*−^ embryo (right). **B,** measure of the area of DFCs cell mass at the 50% epiboly-stage in wt and *msw* ^−*/*−^ embryos. Mean±SE are expressed.

In order to understand the origin of KV defects in M-*msw* ^−/−^ embryos, we decided to analyze KV precursors. KV develops from dorsal forerunner cells (DFCs), a group of cells expressing *sox17* in the most posterior region of the embryos. This group of cells does not undergo involution or epiboly, rather, they reorganize in shape and differentiate in a mature KV containing counterclockwise beating cilia [36], [37]. As the specification of DFCs is complete by the 50% epiboly/shield stage [38], we examined *sox17* expression at that developmental point in WT and M-*msw* ^−/−^ embryos (figure 5A). Results clearly show that the DFCs cell mass present at the margin of epiboly is significantly smaller in M-*msw* ^−/−^ embryos (n = 55) compared to WT (n = 58) (two-sample *t*-test, *t*_(94.762)_ = 9.096; p<0.001; see figure 5B). Conversely, endoderm development seems to be unaffected as embryos grow normally until adulthood and adult fish are fertile. Moreover, we could detect normal expression of endodermal markers as *somatostatin* in the pancreas (data not shown).

### Viewing preference for different stimuli

We then decided to test the hypothesis that a natural occurring polymorphism in a maternal effect allele involved in LR development and lateralization is maintained in the population due to its possible adaptive benefits for predator escape behavior. For this purpose, M-*msw* ^−/−^ embryos were separated according to their parapineal organ position and grown to adulthood.

**Figure 6:**
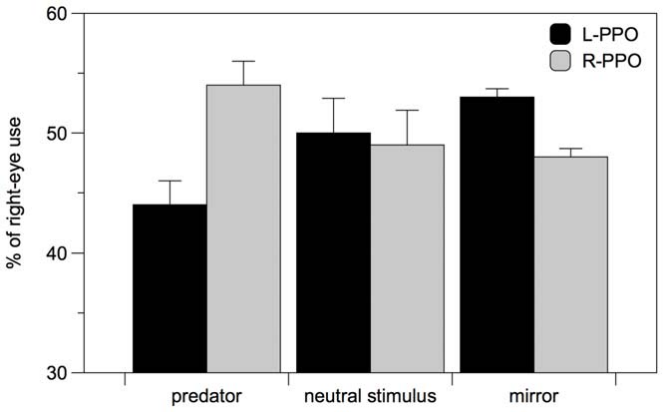
Percentage of right-eye use during the viewing test. Results are presented for the three different stimuli in L- and R-PPO. Means and SE are reported.

L-PPO and R-PPO revealed different patterns of eye use for the both the dummy predator and the mirror image inspection (figure 6). L-PPO looked at the predator preferentially with the left eye (one sample t-test t_(23)_ = 2.596, p = 0.016) while R-PPO use preferentially the right eye (t_(23)_ = 2.742, p = 0.012); the difference between L-PPO and R-PPO is significant (F_(1, 46)_ = 13.499, p = 0.002). When looking at their mirror image L-PPO use preferentially the right eye (t_(23)_ = 2.168, p = 0.041) while R-PPO showed a marginally non-significant tendency to use the left eye (t_(23)_ = 1.756, p = 0.092); the difference between L-PPO and R-PPO is significant (F_(1, 46)_ = 7.691, p = 0.008).

Both L-PPO and R-PPO showed no lateralization in viewing a neutral stimulus (L-PPO t_(23)_ = 1.070, p = 0.296; R-PPO t_(23)_ = 1.420, p = 0.169) and did not differ between themselves (F_(1, 46)_ = 3.146, p = 0.183).

To control for possible effects of sex and order of presentation, we performed a general ANOVA with position of the parapineal organ (L-PPO or R-PPO), sex and order of presentation as between-subjects factors and stimulus type (neutral stimulus, dummy predator and mirror) as within-subject factor. We found a significant interaction between parapineal position and stimulus type (F_(2, 72)_ = 14.860, p<0.001) and no effect of sex (F_(1, 36)_ = 2.606, p = 0.166) or order of presentation (F_(2, 36)_ = 0.714, p = 0.496). No other factors or interactions were significant.

A low laterality index, as observed with mirror image in R-PPO fish or with the neutral stimulus in both groups, may derive either from the subject being aligned in direction but poorly lateralized or from the group being composed of an equal proportion of left and right lateralized individuals. To unravel this point, we calculated for L-PPO and R-PPO an absolute index of laterality (0,5 - | laterality index |), which provides a measure of the degree of lateralization independently from its direction. L-PPO and R-PPO zebrafish differed in the degree of lateralization when viewing the predator (two sample t test t_(46)_ = 2.044, p = 0.047) but not in the other two tests (mirror t_(46)_ = 0.200, p = 0.842; neutral stimulus t_(46)_ = 0.749, p = 0.458). A general ANOVA showed that, on the whole, zebrafish were more lateralized when looking at the predator (repeated measure ANOVA F_(2,92)_ = 33.131; p<0.001); L-PPO and R-PPO did not differ in absolute lateralization (ANOVA F_(1,46)_ = 2.204; p = 0. 145) but there was a significant interaction stimulus x parapineal position (ANOVA F_(2,92)_ = 3,388; p = 0.038).

## Discussion

In the present work we report the evidence of a naturally occurring semi-dominant maternal effect trait affecting left-right development and functional lateralization in zebrafish. Our study demonstrates that the increased frequency of reversed brain asymmetries in the TLRE line, isolated through a behavioral test [21], is due to selection of a maternal effect trait, *mother-of-snow-white*, involved in the early stages of left-right development.

Based on a preliminary Mendelian analysis of this trait, we performed selective crosses to validate the hypothesis that the transmission of the allele reflected those of a recessive maternal effect. Indeed, homozygous recessive females could be identified with the screening of their progeny for the position of the parapineal organ at the 3 dpf stage. Analysis revealed that females could be classified into three groups according to the percentage of reversed brain asymmetries in their offspring and, in addition, the three phenotypic groups of females fit the expected mendelian proportions. In facts, the incross between offspring generated by the outcross of putative *msw-/-* females recreates mendelian phenotypic proportions in females. These two latter points strengths the idea that a major locus is involved in the maternal trait we have isolated. Nevertheless, the variance within each clutch of offspring shows that other genes are possibly involved it the control of the trait considered. Heterozygous females showed an intermediate phenotype. In our hypothesis of a major locus this is the result expected for a semi-dominant allele. However, at this point of our research, we cannot rule out completely a more complex hypothesis such as that of a multilocus trait. Evidence for a maternal effect allele controlling left-right asymmetries has been reported for the direction (also called handedness or body chirality) of shell coiling in snails (*Limnaea* sp.)[18], [19]. In this species body chirality (dextral or sinistral) is determined by a single locus that act maternally [19], [20], [39]. Furthermore, a recent work shows that *nodal* and *Pitx* orthologues have been isolated in two species of snails with opposite direction of shell coiling. The authors found that the side of the embryo that expresses *nodal* and *Pitx* is related to body chirality: both genes are expressed on the right side of the embryo species with dextral shell coiling (*Lottia gigantea*) and on the left side in the species with sinistral coiling (*Biomphalaria glabrata*)[17].

Our data are consistent with the hypothesis of a maternal effect allele controlling the generation of LR asymmetries at the gene expression level. Accordingly, we decided to perform a backward stepwise analysis of key developmental stages of L-R development. Previous work has demonstrated that during L-R development *nodal* activates its own expression and also the expression of its inhibitors of the Lefty/Antivin family [11], [29], [40], [41]. Three nodal-related genes have been isolated in the zebrafish genome: *squint* (*ndr1*), *cyclops* (*ndr2*) [42] and *southpaw* (*ndr3*). In zebrafish the *spaw* gene is the earlier *Nodal* gene with asymmetric transcription along the left lateral plate mesoderm and has been predicted to activate the expression of cyclops (*cyc/ndr2*) and *lefty1* in the left dorsal diencephalon at later somitogenesis stages [30]. In about 50% of M-*msw* ^−/−^ embryos we found altered, bilateral or right-sided, expression of *lefty1* and *spaw* thus reflecting the subsequent organization of neuroanatomical asymmetries. Therefore, embryos expressing *spaw* and *lefty1* on the left side can be predicted to have normal asymmetries, i.e. those with spaw and *lefty1* expression on the right side (reversed asymmetries), while embryos with bilateral expression of *spaw* and *lefty1* can be predicted to develop normal or reversed asymmetries with equal frequencies as already discussed for heart looping by other authors [29]. Further investigations reveal that the *msw* allele is likely to be involved in the differentiation and specification of precursors of the Kupffer's vesicle. In fact, as evidenced by *sox17* analysis, at the 50% epiboly/shield stage M-*msw* ^−/−^ embryos have a smaller KV due to the reduced number of its precursors: the dorsal forerunner cells (DFCs). Events leading to the formation of a reduced or even to an absent KV are known to result in disrupted expression of asymmetric L-R signals as well as subsequent randomization of organ laterality [33]. In line with these evidences, in the present work we argue that the maternally-provided *msw* allele is involved in the mechanism of DFCs differentiation and KV formation without affecting ciliogenesis and cilia motility. Briefly, the maternal effect allele *msw* is reported to control LR development of epithalamic structures by regulating KV morphogenesis.

Our findings point out the evolutionary importance of maternal genetic control on the behavioral phenotype of the progeny. In fact we hypothesize that in zebrafish, as in other lower vertebrates with external fertilization and lack of parental/maternal care, the main source for genetic control of phenotypic plasticity and evolutionary response to selection could be represented by the maternal genetic factors provided in the egg [43]. Maternal factors are known to drive early stages of embryonic development before the activation of the zygotic genome [44]. For example, in the snail in *Lymnaea stagnalis* the direction of body chirality, which is controlled by a maternal effect gene [19], [20], also correspond to an asymmetry in the brain that is correlated with lateralized precopulatory behaviors [45].

To support the hypothesis that the msw allele controls lateralized behaviors related to diencephalic asymmetries, we analyzed left-and right-parapineal M-*msw* ^−/−^ fish in a “viewing test”. The results of the viewing tests in fish of the F3 generation are largely consistent with the pattern of lateralization already described in a previous study using different laterality tests on fish of the *msw* pedigree (generation F1) [24]. L-PPO and R-PPO showed opposite eye preference when scrutinizing a model predator. Moreover these two groups showed different eye preference when the stimulus was a mirror while no preference was observed using a neutral stimulus. In other words, there is a complementary eye preference related to the nature of the stimulus, as already reported in other studies [22], [23], [24]. However, there are some potential disadvantages in possessing an asymmetrical perceptual system. Since the physical world is neutral to left and right, any lateralized deficit might leave an animal vulnerable on one side or unable to attack on the other side [46], [47]. Indeed, one might expect that natural selection would prevent the fixation of a single phenotype while causing left- and right-type individuals to occur in equal proportions in a given species. In fact, lateralization at the population level (i.e., the alignment of the direction of lateral biases in most individuals within a population) is quite common in a wide range of vertebrates [47]. To disentangle this point Vallortigara and Rogers suggest that what is advantageous for an individual depends on what the other individuals of the group are doing [2], [80] and lateralization at the population-level may have evolved as an “evolutionarily stable strategy” (ESS) to coordinate behavior among individuals. On the other hand, a percentage from 10% to 35% of individuals do not conform to the pattern of the majority of the population [48]. Ghirlanda and Vallortigara [49] suggest that an advantage for the minority group depends on the frequency of these individuals (i.e., an advantage that disappears when these individuals increase in number). For the example of escape response, while the majority of prey gain protection by keeping together, a minority gain the same escape probability by trading off protection from the group in favor of an advantage (i.e. unpredictability) in the face of predators [49]. Thus, results of the viewing test can be interpreted considering these arguments. For example, when the simulated predator was visible, the most common phenotype (L-PPO) showed a left-eye preference and presumably gains protection by keeping together. Conversely, the less frequent phenotype (R-PPO), showed a right-eye preference and, presumably, enjoys an advantage because its behavior is less predictable by the predator. But what is the role of the maternal effect allele in this framework? One possible explanation is that maternal effects might be related to the long term maintenance of this evolutionary stable strategy in zebrafish because maternal effect alleles are partially hidden to selection [50].

Numerous studies have dealt with the genetics of lateralization, focusing their attention in mammals and other vertebrates [2]. In humans the most notable example of lateralization is handedness, with the great majority of individuals (approx. 90%) right-handed [51]. This is mainly based on the distribution and genetic modeling of handedness in humans. Handedness is heritable and 7.6% of the children of two right-handed parents are left-handed (this percentage increases to 19.5% if one of the parents is left-handed and to 54.5% if both the parents are left-handed [52]. Many studies propose single-locus models to investigate the genetic basis of handedness. In these models one allele specifies right-handedness, whereas another allele specifies left- or right-handedness at random [53], [54], [55]. These models propose the existence of alleles for right-handedness (in combination with left-hemispheric dominance for language), and that the direction of handedness (and language) is generated by chance.

Recently Klar [56] demonstrate that hand preference can be linked to the directionality of scalp hair-whorl rotation. The author also proposes a model (“random-recessive model”) where a single gene with two alleles controls both handedness and hair-whorl orientation so that the single dominant gene causes right-handedness and clockwise whorl rotation in the dominant homozygous and heterozygous situations, and the recessive and nonfunctional allele confers a statistical random chance in recessive homozygosis [57]. Anticlockwise hair-whorl direction has been related to an increased probability of non-right-handedness and atypical (right) hemispheric language dominance. Jansen and collaborators [58] also investigated the relationship between scalp hair-whorl direction, handedness and hemispheric language dominance but they found no association. Despite these findings there are some problem with these models. For example in several twin studies, the comparison between monozygotic and dizygotic twins revealed no difference in the incidence (from 10 up to 25%) of left-handedness [52], [59], [60]. A second problem is that males show higher incidences of left-handedness (11.6%) compared to females (8.6%) [54].

Evidence also comes from studies of non-human species. In chimpanzees, handedness is usually measured by means of a tube task where the subject is requested to obtain peanut butter from a tube using one hand. The percentage of right-handed offspring from right-handed mothers ranged from 46 to 86% as a consequence of developmental instability experienced by the offspring indicating that other mechanisms, rather than simple mendelian genetic factors, affect lateralization [61]. On the other hand although mice can be selected for the degree of lateralization, selective breeding for the direction of pawedness were not successful [62]. In order to overcome the problems raised by simple genetic models, the classical argument that environmental factors can largely influence the degree or strength of lateralization in both humans and other vertebrates has been recently suggested [63]. However, another possibility worth considering is that non-Mendelian genetic mechanisms are involved in control of laterality and, while confounding genetic analysis of offspring phenotypes, these might introduce a higher level of complexity in adaptation and evolution. In fact, our finding that the maternal genotype can influence the lateralized behavior of offspring might explain some of the difficulties experienced by research in mammals. In mice, the embryonic genome is activated from the late stage of 1-cell zygotes and becomes dominant in the 2-cell stage embryos, while in humans, it is activated between the 4- and the 16-cell stage [64], [65]. Therefore, in Eutherians maternal genetic factors of the oocyte can only play limited genetic effects as the molecular contribution of maternal factors is limited to a small amount of components provided in the egg degraded shortly after fertilization [66], [67]. Indeed, despite their putative important functions, especially during the oocyte to embryo transition, few mammalian “in ovo” maternal-effect genes have been identified [68], [69]. However, due to the prolonged maternal care, it is tempting to speculate that others, more indirect, maternal genetic controls can be acting on the progeny's behavior. In fact it has been already demonstrated that two inbred mice strains C57BL/6J and BALB/cJ differing for high and low levels of maternal cares, respectively, exerts a strong differential influence on stress and anxiety-like behavior of the progeny [70], [71].

In conclusion, as maternal effect genes are expected to evolve more rapidly when compared to zygotic ones [50], [72] they must be seriously considered as a gold mine in behavioral ecology and adaptation studies. Finally, given that phenotypes caused by maternal effect alleles are detected in the progeny, their isolation and study might be facilitated in animals with a fully innate behavioral setup

## Materials and Methods

### Fish lines

Wild type stocks used in this work and for selection are from Giotto Leo (GT that comes from a pet shop in Padova and then bred in laboratory conditions for several generations) or from Tupfel Longfin (TL) strain. *msw* and wt Zebrafish strains [21], [24] were maintained under standard conditions and staged as previously described [73], [74]. Once raised to adulthood F_2_ females were housed separately to allow their identification. For behavioral tests *msw* ^−/−^ females were mated to males from the transgenic line tg(*foxD3*:GFP)zf15 [28]. Embryos were placed in Petri dishes provided with embryo medium [74] and incubated at a constant temperature of 28°C. Between 28 somite and prim 5, embryos were treated with a 0.003% 1-phenyl 2-thiourea (PTU) solution that inhibits melanogenesis by blocking all tyrosinase-dependent steps in the melanin pathway [75]. This procedure allows us to improve signal detection by expression of GFP. At 3days post-fertilization GFP-expressing larvae were anesthetized in Tricaine solution (3-amino benzoic acid ethyl ester provided by Sigma) and analyzed using a stereo microscope (Leica MZFLIII) equipped with a UV-lamp. The whole sample of larvae was then divided and raised according to the position of the parapineal organ into two groups: L-PPO (subjects with the parapineal organ on the left side) and R-PPO (subjects with the parapineal organ on the right side). All animal work has been conducted according to relevant national and international guidelines. The work has been approved by the ethic committee of the University of Padova with the ID 19-2010.

### Whole-mount in situ hybridization

Whole-mount in situ hybridization was performed as described previously [76]. Reagents were obtained from Roche and Sigma unless indicated otherwise. Labelled RNA antisense probes were synthesized using UTP-digoxigenin. Probes were incubated at 65°C for *lov* [26], *sox17* [77] and *ntl* [78], at 62°C for *lefty1* [40] and 69°C for *spaw* [30] in hybridization solution containing 60% formamide. Embryos were stained using NBT-BCIP (Sigma) as substrate.

### Fluorescent beads injection

This procedure has been performed according to protocols described in previous works of other authors [13], [35]. Fluorescent beads of 0.5 µm diameter (Polysciences) were diluted 1∶100 in dd water and 0.1% of Phenol Red (Sigma) and then injected into KV of live embryos at 8–10 somite stage. Embryos were manually dechorionated, placed into 3×3 mm wells of injection plates made in 0.8% agarose in Ringer's solution on coverslips and mounted in 1% low melting agarose (Sigma) with the KV facing upwards. Beads movement was visualized using a 40x/0.80 water immersion lens and recorded on a Nikon fluorescence microscope (Nikon ECLIPSE 80i) using ATI Multimediacenter software.

### Bright field screening of Kupffer's vesicles

Embryos at the 5–10 somite stage were mounted and microinjected as previously described [34]. Vesicles were visualized using a 40x/0.80 water immersion lens on a Nicon ECLIPSE 80i microscope and measured with the aid of the software Image Pro Plus 6.0.

### Analysis of DFCs

The area of DFCs cell mass after in situ hybridization has been measured as depicted in Figure S2: 8bit images as in Fig. 5 have been imported in ImageJ, inverted (Edit/Invert) and their threshold has been set to 220 (Image/Adjust/Threshold). The stained area of the KV's has been measured (Analyse/Analyse particles) after the scale of the figure was set (Analyse/Set Scale) using a Burker's chamber. In a few cases we found a disaggregated DFC cell mass and this was measured as the sum of smaller cell masses.

### Viewing preference for different stimuli

Viewing tests have been successfully used in previous studies to estimate asymmetries in eye use in fish [25], [79]. Aim of this experiment was to measure lateralization in eye use for stimuli that differ qualitatively.

Twenty-four adult zebrafish with left parapineal (L-PPO; 12 females and 12 males) and 24 adult zebrafish with right parapineal (R-PPO; 12 females and 12 males) were used for this experiment.

**Figure 7:**
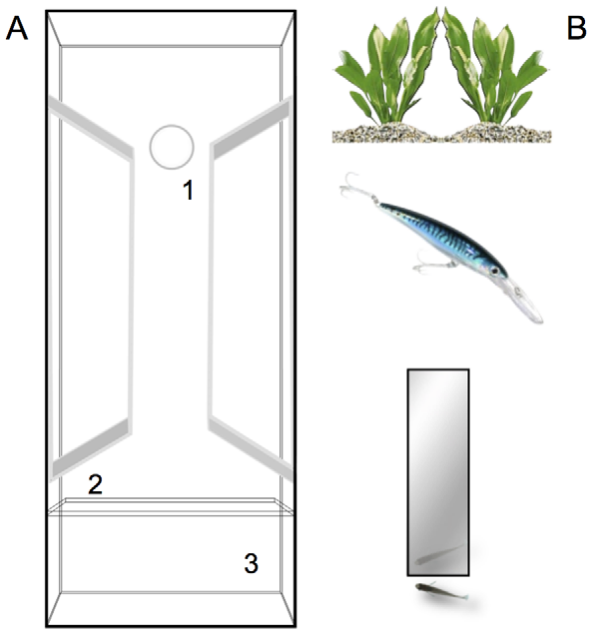
The viewing test. **A,** Schematic representation of the apparatus for the viewing test. 1 indicates the swimway, 2 indicates the glass partiton and 3 indicates the stimulus area. **B**, stimuli used in the test. From above; the neutral stimulus, the model predator and the mirror

The apparatus used was similar to that described elsewhere [25]. Briefly, it consisted in a large glass tank (19×72 cm, 32 cm in height) with a ‘swimway’ in the middle (figure 7A) (35×6.5 cm). At one end there was a glass partition (18.5×7×32 cm) that delimited a “stimulus area” containing the target stimulus. Subjects were exposed to three different stimuli (figure 7B): a neutral stimulus that consisted in the reproduction of a plant, *Echinodorus bleheri*. To prevent any side bias the original picture was duplicated and then rotated horizontally in order to obtain a symmetric stimulus. The second stimulus, the hazardous one, was a dummy predator, previously adopted for similar purposes in other species of small fish [25]. The third stimulus was a small mirror (6×30 cm) positioned in the center of the stimulus area. As for the dummy predator, mirrors have been shown to elicit a social response in small fish and in zebrafish as well [24]. An opaque cylinder (3 cm in diameter) at the opposite end prevented sight of the stimuli before the fish entered the swimway. Each fish were tested singly in the apparatus and the entire session was recorded by means of a videocamera mounted above the swimway.

Six identical apparatus were provided. Fish were housed singly into the apparatus for a 5-day period. During this period subjects were fed twice a day with *Artemia salina* nauplii and each apparatus was lit for 12 h a day (using a single 58W fluorescent lamp), in order to make the test fish familiar with the novel environment (no target was present in this phase).

The experiment has been completed in three sessions spaced 3 h apart. Two fish (1 R-PPO and 1 L.PPO) were first tested with the neutral stimulus, two fish with the dummy predator and the remaining two fish with the mirror as target. The behavior of the fish was recorded from its entering the swimway for 5 min. The original video recordings were subsequently edited (Adobe Premiere® Pro2.0) in order to obtain 5 fps clips. For each frame, fish positions were scored using a computer program (written in Delphi 4 – Borland®) that calculated the degree of alignment (angle in degrees formed by prolongation of the major body axis of the fish with respect to the glass partition) between the subject and the glass partition. Data were discarded when the fish was perpendicular to the partition (binocular stimulation) or when it formed an angle larger than 90° with respect to the partition. From the analysis of the recordings we derived an index of eye preference as:

[(frequency of right-eye use):(frequency of right-eye use + frequency of left-eye use)].

## Supporting Information

Figure S1 ***leftover*** **expression in embryos from WT females.** The graph represents the percentage of right *lov* expression in crosses of WT females with either GTLE or WT males. Mean and SD are espressed.
(TIF)

Figure S2 **Calculation of DFC's area.** 8bit images as in Fig. 5 have been imported in ImageJ, inverted and their threshold has been set to 220. The stained area of the KV's has been measured after the scale of the figure was set using a Burker's chamber.
(TIF)

Movie S1 **Normal leftward flow inside the KV in M** ***msw*** **^+/+^** **embryos.** Dorsal view inside the KV of a live 10-somite stage M*msw^+/+^* embryo. Fluorescent beads injected into the KV show a net counterclockwise movement.
(MOV)

Movie S2 **Leftward flow inside the KV in wt** **embryos.** Dorsal view inside the KV of a live 10-somite stage wt embryo. Fluorescent beads injected into the KV show a net counterclockwise movement, evidencing no difference compared to M*msw^+/+^* embryos.
(MOV)
