# Supplementary figures and images for: Isolation and Genetic Characterization of Mother-of-Snow-White, a Maternal Effect Allele Affecting Laterality and Lateralized Behaviors in Zebrafish
Source: PLoS One. 2011 Oct 13;6(10):e25972. doi: 10.1371/journal.pone.0025972 (PMC3192786; doi:10.1371/journal.pone.0025972)

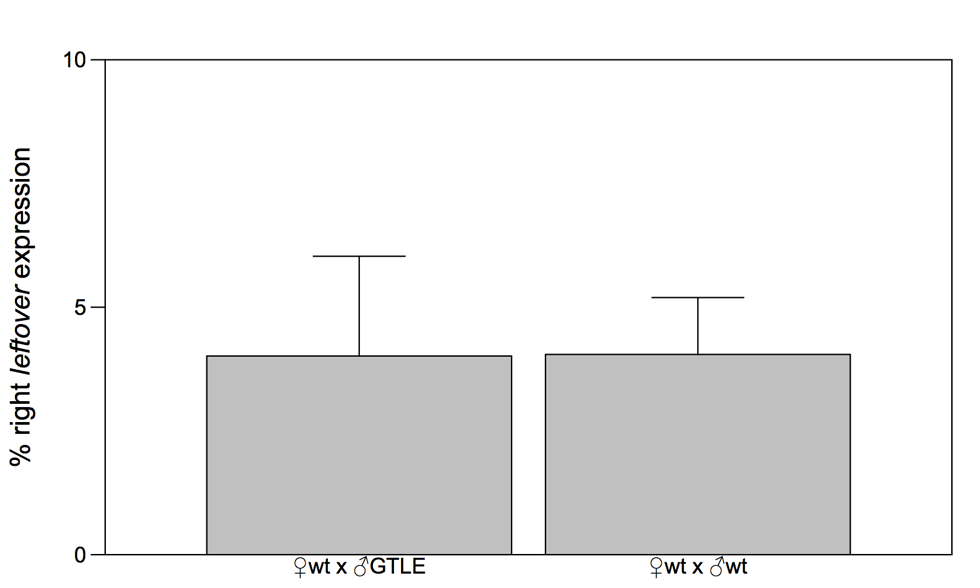

Supplement: Figure S1 — leftover expression in embryos from WT females. The graph represents the percentage of right lov expression in crosses of WT females with either GTLE or WT males. Mean and SD are espressed. (TIF) [file pone.0025972.s001.tif]

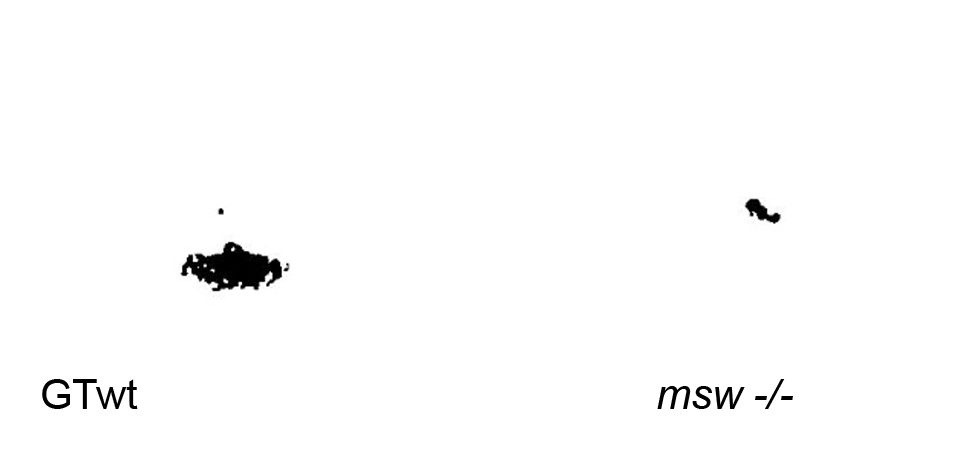

Supplement: Figure S2 — Calculation of DFC's area. 8bit images as in Fig. 5 have been imported in ImageJ, inverted and their threshold has been set to 220. The stained area of the KV's has been measured after the scale of the figure was set using a Burker's chamber. (TIF) [file pone.0025972.s002.tif]
